# Medical management of primary hyperparathyroidism

**DOI:** 10.20945/2359-3997000000558

**Published:** 2022-11-10

**Authors:** Francisco Bandeira, Janiere de Moura Nóbrega, Lucian Batista de Oliveira, John Bilezikian

**Affiliations:** 1 Universidade de Pernambuco Faculdade de Ciências Médicas Hospital Agamenon Magalhães Recife PE Brasil Divisão de Endocrinologia e Diabetes, Hospital Agamenon Magalhães, Faculdade de Ciências Médicas, Universidade de Pernambuco, Recife, PE, Brasil; 2 Universidade de Pernambuco Hospital Agamenon Magalhães Faculdade de Medicina Divisão de Endocrinologia, Diabetes e Doenças Ósseas Recife PE Brasil Divisão de Endocrinologia, Diabetes e Doenças Ósseas, Hospital Agamenon Magalhães Faculdade de Medicina, Universidade de Pernambuco (UPE), Recife, PE, Brasil; 3 Columbia University College of Physicians & Surgeons Metabolic Bone Diseases Unit New York NY USA Division of Endocrinology, Metabolic Bone Diseases Unit, College of Physicians & Surgeons, Columbia University, New York, NY, USA

**Keywords:** Primary hyperparathyroidism, hypercalcemia, parathyroid hormone, conservative therapies

## Abstract

Primary hyperparathyroidism (PHPT) is an endocrine disorder resulting from the hyperfunction of one or more parathyroid glands, with hypersecretion of parathyroid hormone (PTH). It can be managed by parathyroidectomy (PTX) or non-surgically. Medical therapy with pharmacological agents is an alternative for those patients with asymptomatic PHPT who meet guidelines for surgery but are unable or unwilling to undergo PTX. In this review, we focus upon these non-surgical aspects of PHPT management. We emphasize the most studied and widely used pharmacological alternatives: bisphosphonates, denosumab, cinacalcet and hormone therapy, in addition to combined therapy. We also address the relevant aspects of perioperative management.

## INTRODUCTION

Primary hyperparathyroidism (PHPT) is an endocrine disorder characterized by excess secretion of parathyroid hormone (PTH), resulting from the hyperfunction of one or more of the parathyroid glands. A single, benign parathyroid adenoma is the most common presentation (1,2). Biochemical hallmarks are hypercalcemia and high or inappropriately normal PTH (1–3). More recently, normocalcemic PHPT has been described, expanding the clinical phenotype of the disease. In this form, normal adjusted total serum and normal ionized calcium levels are consistently observed along with frankly elevated levels of PTH, in the absence of any other cause for a high PTH concentration (1,4).

The definitive therapy of PHPT is parathyroidectomy. However, patients who meet a surgical guideline may not be willing or medically able to undergo parathyroid surgery. Thus, pharmacological management is an important alternative in this setting (2,3,5).

This review focuses on the medical management of PHPT.

## WHEN TO CHOOSE MEDICAL TREATMENT

In patients with symptomatic PHPT, defined by clinical involvement of target-organs (renal and skeletal), parathyroidectomy (PTX) is recommended (2,3,5,6). In patients with asymptomatic disease, surgery is also recommended if any one of the following is present: serum calcium concentration of >1 mg/dL above the upper normal limit; T-score <–2.5 at the lumbar spine, femoral neck, total hip or distal one-third of the radius; presence of vertebral fractures by an imaging modality; creatinine clearance of <60 mL/min, kidney stones or nephrocalcinosis by abdominal imaging, marked hypercalciuria and/or increased risk of kidney stones by biochemical stone risk analysis, and the guidelines also recommend surgery in those aged <50 years (3). For those patients with asymptomatic PHPT who do not meet guidelines for surgery or who do but are unable (e.g. very elderly people with multiple comorbidities) or unwilling to undergo PTX, alternative, non-surgical become more relevant (3,5,6).

## CALCIUM AND VITAMIN D

Normal calcium intake is recommended, similar to that indicated for the general population (3). The Institute of Medicine provides reasonable guidance of 1,000-1,200 mg from all sources daily (7). A restriction in the intake of calcium can lead to further stimulation of PTH and further progression of the disease (8).

Another important nutrient is vitamin D. In PHPT, vitamin D deficiency can be associated with increased PTH levels and further progression of the disease (1,3,9,10). Vitamin D supplementation in vitamin D-deficient patients will restore levels and reduce PTH. When vitamin D is administered prudently, in modest doses, (e.g. 1,000 IU/day) worsening hypercalcemia is rarely seen (10–12). 25-hydroxyvitamin D (25OHD) levels should always be maintained >20 ng/mL, but some experts recommend levels of >30 ng/mL, in PHPT (3,9). The reason for this recommendation for a higher threshold of 25OHD is that when levels are <30 ng/mL, further increases in PTH have been observed. (13).

## ANTIRESORPTIVE DRUGS

Oral bisphosphonates (BP) are effective in improving bone mineral density (BMD) in the lumbar spine (LS), femoral neck (FN) and total hip (TH). The distal 1/3 radius is not reliably increased. Bone turnover markers fall. The serum calcium does not change. There are no data on fracture risk reduction (1,3,14–19). There is no robust evidence regarding the use of intravenous BP in the clinical treatment of PHPT.

A randomized placebo-controlled clinical trial, which enrolled 44 patients with asymptomatic PHPT, demonstrated that the use of alendronate (ALN) for 2 years provided significant increases in BMD of the LS (+6.85%; P < 0.001), TH (+4 .01%; P < 0.001) and FN (+3.67%; P = 0.038), with no significant changes in BMD of the distal radius. In addition, ALN led to marked reductions in bone turnover markers, with a 66% reduction in urinary N-telopeptide (NTX) in the first 3 months and a 49% reduction in bone-specific alkaline phosphatase (BSAP) in 6 months. Serum and urinary calcium and PTH levels were not changed by ALN (14). Post hoc analysis of this study, which sought to assess the response to one-year bisphosphonate therapy in men with PHPT, demonstrated results similar to those seen in postmenopausal women (16). Other randomized studies have demonstrated improved BMD of the spine and hip with ALN in postmenopausal women (17) and in the elderly (18).

Risedronate is another bisphosphonate that has been studied in the management of PHPT. LS BMD increased significantly (+5.62% at 2 years), with FN and TH BMD remaining stable (+1.18% and +1.68%, respectively). An observational study that included 32 postmenopausal women with PHPT (16 undergoing PTX and 16 treated with 35 mg risedronate for 2 years) evaluated volumetric BMD and the trabecular cortical area of the tibia by peripheral quantitative computed tomography (pQCT). It demonstrated the superiority of PTX in increasing trabecular bone mineral content and volumetric BMD (+6.44% and +4.64%, respectively) compared to risedronate (-0.14% and +0.24%, respectively). In cortical sites, there was no significant change after PTX, although the percentage change in cortical volumetric BMD was greater with PTX (0.39% vs. -0.26%, p < 0.05) (19).

In addition to bisphosphonates, denosumab has emerged as an antiresorptive option for PHPT (20,21). A retrospective longitudinal study evaluated the effects of denosumab on bone health in 50 elderly women, of whom 25 had PHPT and 25 had postmenopausal osteoporosis. After 2 years of denosumab use, women with PHPT showed greater bone mass gain in LS, FN, and TH than those with postmenopausal osteoporosis (20). Another retrospective study comparing PTX with denosumab showed a dissociation between outcomes by BMD and the trabecular bone score (TBS). PTX was associated with a more significant improvement in BMD of the LS and TH, while changes in TBS favored treatment with denosumab (21). This same study showed that the drop in serum calcium levels, expected after administration of denosumab, was not sustained one year after starting its use (21).

## ESTROGEN THERAPY AND SELECTIVE ESTROGEN RECEPTOR MODULATORS (SERMS)

Hormone replacement therapy (HRT) has been shown to improve BMD, suppress bone turnover, and reduce calciuria in postmenopausal women with asymptomatic PHPT (22,23). A double-blind, randomized clinical trial, which compared HRT (conjugated estrogens, 0.625 mg/day, and medroxyprogesterone, 5 mg/day) with placebo, demonstrated that HRT for 2 years provided BMD gains in LS (+5.2 ± 1.4%; P = 0.02), FN (+3.4 ± 1.5%; P = 0.05) and in the total body (1.3 ± 0.4%; P = 0.004), in addition to reducing serum alkaline phosphatase levels by 22%, urinary excretion of hydroxyproline by 42%, urinary NTX by 54% and calciuria by 45%. There were no significant changes in serum calcium and PTH levels (22). A 2 to 4 year extension of this study, which involved 23 of the 44 women previously included, demonstrated an additional incremental effect on bone density with HRT and continued control of bone turnover markers (23). There are no data directly evaluating fracture reduction with HRT in PHPT (24).

SERMs have also been studied as a possible option in the treatment of asymptomatic PHPT in postmenopausal women. In an 8-week randomized placebo-controlled clinical trial, the use of raloxifene at a dose of 60 mg/day was associated with a reduction in serum calcium and bone turnover markers (osteocalcin and serum NTX). No significant changes were observed in serum PTH and calciuria (25).

## CINACALCET

Cinacalcet is a calcimimetic agent that lowers serum calcium and PTH levels by binding to calcium sensitive receptors (CaSR) in the parathyroids (2,26).

In a one-year multicenter, randomized, double-blind, placebo-controlled study, 78 patients with PHTP were randomized to cinacalcet (30-50 mg twice daily) or placebo. Seventy-three percent of patients in the cinacalcet group achieved the primary endpoint, namely normal serum calcium level (≤10.2 mg/dL or 2.57 mmol/L) with a reduction of at least 0.5 mg/dL from baseline. By direct comparison, only 5% in the placebo group met this endpoint (P < 0.001) (27). Additionally, cinacalcet reduced serum levels of PTH and increased bone turnover markers, but without changes in BMD. In an open-label extension, these results were maintained over a 5-year period (28).

A more recent 28-week randomized clinical trial with 67 PHPT subjects found that normocalcemia was achieved in 75.8% of patients treated with cinacalcet (initial dose of 30 mg twice a day, with sequential titrations, reaching up to 90 mg three times a day) versus 0% of those who received placebo (P < 0.001) (29).

In an open-label, single-arm study, 17 patients with intractable PHPT and serum calcium greater than 12.5 mg/dL were treated with cinacalcet. The serum calcium fell by 1 mg/dL or greater in 15 patients (88%). Another 15 patients experienced treatment-related adverse events, none of which were serious. The most common adverse events were nausea, vomiting, and paresthesias (30). These adverse reactions were uncommon and dose-dependent (1,2,29).

## COMBINATION THERAPY

Combination therapies of cinacalcet with ALN and of cinacalcet with denosumab have been studied in comparison to monotherapy (31,32). A retrospective study evaluated the responses of cinacalcet in monotherapy (n = 13) and in combination with ALN (n = 10), showing improvements of serum calcium, phosphorus and PTH, as well as in urinary calcium, in both groups, with improvement in BMD only in the group treated with the combination (increases of 9.6% in the LSBMD and 3.9% in the THBMD) (31). In a double-blind randomized trial, 45 patients with PHPT were randomly allocated to receive cinacalcet plus denosumab (n = 14), denosumab plus placebo (n = 16) or placebo plus placebo (n = 15). BMD significantly improved, both in the hip and spine, in the two groups that received denosumab. Sustained normalization of calcium levels occurred in 63.8% of the group that received cinacalcet, but was not observed in the other two groups (32). Table 1 presents the effects of drug treatment of PHPT.

**Table 1:** The effects of medical therapy of primary hyperparathyroidism

Treatment	BMD	BTM	Serum calcium	Serum PTH
Alendronate	Increases	Decreases	No change	No change or transient increases
Zoledronic acid	Increases	Decreases	NA	NA
Cinacalcet	No change	Increases	Decreases	Decreases
Denosumab	Increases	Decreases (transient?)	Decreases	Increases
Denosumab + Cinacalcet	Increases	Decreases	Decreases	Transient increases
Conjugated estrogens, and medroxyprogesterone	Increases	Decreases	No change	No change
Raloxifene	NA	Decreases	No change	No change

BMD: bone mineral density; BTM: bone turnover markers; PTH: parathyroid hormone; NA: not available.

## PERIOPERATIVE MEDICAL TREATMENT

Medical treatment of PHPT can also be started when there is a surgical indication, either before or after PTX. A retrospective study comparing BMD 1 year after PTX alone (n = 24) with PTX plus bisphosphonate combination treatment (n = 26) observed significant increases in bone mass in both groups, but the increase in FN-BMD was higher in the PTX-only group (P = 0.011). In addition, a significant association was found between changes in serum alkaline phosphatase levels and FN-BMD, in the PTX only group, demonstrating that combined BP treatment can interfere with gains in bone mass provided by PTX(33). In contrast, a cohort study with 1,737 individuals with PHPT evaluated the effect of adding bisphosphonates before or after PTX on bone health. PTX alone and bisphosphonate use followed by PTX were associated with a reduction in fracture risk. It suggests that bisphosphonate use before PTX does not interfere with bone density improvement after PTX (15). Likewise, the preoperative use of bisphosphonates in patients with severe bone involvement – *osteitis fibrosa cystica* – appeared to reduce the severity of the postoperative bone hunger syndrome, without preventing a significant increase in BMD(34). In one study, zoledronic acid (ZOL) was used in a randomized, placebo-controlled trial on 56 patients with PHPT and osteoporosis after PTX. There were significant increases in BMD of LS (P = 0.039 and 0.017 for T and Z scores, respectively) and FN (P = 0.045 for Z score), as well as reductions in bone turnover markers in the ZOL group compared to placebo. Thus, an increase in BMD was demonstrated after PTX with and without ZOL, but this effect was significantly greater with the use of ZOL postoperatively (35).In a small case series, denosumab was used to normalize serum calcium levels before PTX in four patients with severe hypercalcemia, demonstrating safety and efficacy (36).

It is worth noting that there are no differences in preoperative clinical management in patients with genetic disorders, such as multiple endocrine neoplasms. The operative approach, which may differ, is a function of the preoperative management (3).

## FOLLOW-UP OF MEDICAL MANAGEMENT

Annual measurement of serum calcium, PTH and creatinine is recommended, with calculation of the estimated glomerular filtration rate. BMD should be monitored every 1-2 years. Follow-up imaging such as spine X-ray or vertebral fracture assessment (VFA) should be performed if there is height loss, new back pain, of other clinical concerns. Imaging tests to search for kidney stones should be performed based on clinical suspicion (1–3). Long term conservative management of PTH can be associated with worsening bone density, particularly after 10 years (3).

In conclusion, although PTX is the only definitive treatment for PHPT, medical therapy can be considered in patients who meet criteria for surgery but in whom it is refused or medically contraindicated. To increase BMD, BPs are effective. To reduce the serum calcium cinacalcet is effective.
